# Manifesto: challenging the standard cosmological model

**DOI:** 10.1098/rsta.2024.0036

**Published:** 2025-02-13

**Authors:** James Binney, Roya Mohayaee, John Peacock, Subir Sarkar

**Affiliations:** ^1^ Rudolf Peierls Centre for Theoretical Physics, University of Oxford, Oxford OX1 3PU, UK; ^2^ Sorbonne Universite, CNRS, Institut d'Astrophysique de Paris, 98bis Bld Arago, Paris 75014, France; ^3^ Institute for Astronomy, University of Edinburgh, Royal Observatory, Edinburgh EH9 3HJ, UK

**Keywords:** cosmology, dark energy, large-scale structure, standard model, universe

## Abstract

We outline the rationale for holding a Discussion Meeting thus titled at the Royal Society, London during 15 and 16 April 2024, and summarize what we learnt there.

This article is part of the discussion meeting issue ‘Challenging the standard cosmological model’.

## 1. Synopsis of the meeting

Is the Universe simple enough to be adequately described by the standard Lambda–Cold–Dark–Matter (ΛCDM) cosmological model, which assumes the isotropic and homogeneous Friedmann–Lemaitre–Robertson–Walker (FLRW) metric? Tensions have emerged between the values of cosmological parameters estimated in different ways. Do these tensions signal that our model is too simple? Could a more sophisticated model account for the data without invoking a cosmological constant? Individual items of conflicting evidence sit in different parts of the literature and there is a danger that they are underappreciated in the cosmology community because they do not fit into a consistent picture. This provided us with the motivation for bringing together in one place the diverse observations and puzzles that challenge the standard model, in the hope that this might illuminate which challenges to take most seriously.

### (a) Why this was an appropriate topic

The interpretation of any measurement is implicitly subject to model assumptions. Contemporary cosmology assumes that the Universe emerged from the Big Bang almost perfectly homogeneous and isotropic and that the structure we now see emerged from a Gaussian random field of tiny fluctuations. The programme of ‘precision cosmology’ is to measure the parameters that quantify this underlying perfectly symmetric model and the superposed field of fluctuations.

In recent years, inconsistencies have arisen between the values extracted from different data sets [1–4]. For example, the value of the Hubble parameter inferred from observations of the cosmic microwave background (CMB) is inconsistent with that measured locally. Similarly, the clustering parameter $S_{8}$ inferred from the CMB is inconsistent with that inferred from weak gravitational lensing. A further issue is that the streaming velocities of the galaxies around us with respect to the CMB seem to show no signs of abating out to several hundred megaparsecs; this calls into question the assumption that the streaming was generated by the gravitational acceleration field associated with the field of large-scale density fluctuations. Such inconsistencies may signal that the Universe is not as simple as cosmologists assume, in which case it cannot be fully described by the model parameters we have been ‘measuring’.

Our aim was therefore to gather leading cosmologists to re-examine the fundamental assumptions of isotropy and homogeneity that underlie the standard cosmological model. On what length scale does the assumed statistical homogeneity of the Universe actually emerge? What are the implications of the observed mismatch between the rest frames in which the CMB and distant matter (traced by radio galaxies and quasars) are isotropic? If this mismatch is a real effect, can the data for distant supernovae be successfully modelled without appealing to a cosmological constant? Is the cosmological constant Λ, the dominant constituent of the Universe in the standard cosmological model, even consistent with quantum gravity? The current ‘concordance’ ΛCDM standard model of cosmology intertwines many untested assumptions with observational data. Our intention was that experts would critically discuss these issues in the light of recent data and ask whether or not the time has come to move beyond the standard cosmological model.

As Einstein remarked, ‘We cannot solve our problems with the same level of thinking that created them’. The goal of this meeting was to explore the limits of our approximations to cosmology and to plan how best to use the flood of data expected in the near future from, e.g. the Dark Energy Spectroscopic Instrument (DESI) and the Rubin Observatory Legacy Survey of Space and Time, to build a better cosmological model.

### (b) Meeting objectives

The meeting was structured to bring together general relativists and cosmologists, astronomers and particle physicists to discuss and debate the validity of the standard cosmological model, according to which the dominant component of the Universe is Einstein’s cosmological constant. The latter is often interpreted as the ground state energy of the quantum field theoretic vacuum, but the vacuum’s energy density is known to be at least 60 orders of magnitude larger than the effective density required by observations, so at the level of quantum fluctuations, the expectation of the energy-momentum tensor cannot be what is required on the right-hand side of Einstein’s equations. Hence, the ΛCDM model lacks a fundamental physical understanding, even though it is phenomenologically highly successful in giving a close match to a wide variety of data. It has been suggested that Λ-dominated de Sitter space–time may even be in conflict with fundamental notions such as the unitarity of the S-matrix in a quantum theory of gravity [5]. The objective of this meeting was to air and debate these concerns and to ask whether a less problematic interpretation of the data could be synthesized if we drop some of the strong but rarely articulated assumptions inherent in the standard cosmological model.

In 1987, George Ellis and collaborators discussed the ideal observational data that would enable one to determine the space–time structure of the observable Universe. Their paper on the ‘Cosmological Fitting Problem’ [6] articulated the model-based assumptions and demonstrated their effect on the interpretation of the data. However, despite its clear principles, this methodology has not attracted much attention in the community. The tensions that have arisen in recent years with the advent of ‘precision cosmology’ may well be manifestations of this fitting problem, that is, the consequence of interpreting data for the real Universe in the framework of the isotropic and homogeneous FLRW model. The Universe is neither exactly isotropic nor homogeneous and some think that the ‘backreaction’ from spatial averaging could mimic a cosmological constant term [7]. Another approach is to note that we are not Copernican but ‘tilted’ observers, embedded as we are in a deep bulk flow of numerous galaxies and that consequently we may be misinterpreting global deceleration of the Hubble expansion rate as a local acceleration [8]. Analysis of the Hubble diagram of supernovae has been claimed to lend support to this conjecture.

Enormous resources are currently being devoted to efforts to characterize dark energy, but how sure are we that dark energy is real? This meeting was intended to prompt the community to examine this question, which is a critical issue for both fundamental physics and observational astronomy.

### (c) Why this meeting was different

Over the last 30−40 years, cosmology has developed into a large-scale industry that employs thousands of physicists and astronomers. Large observational programmes have accumulated data on the CMB, the intergalactic medium, the intergalactic gravitational field and the density of galaxies, quasars and radio sources. Each of these endeavours gives rise to numerous international meetings to discuss how to gather the data and how to analyze them. But, the analysis is invariably in the context of the standard cosmological model. Some results do not quite fit, but in any one field, tensions may be dismissed as insignificant. By bringing together workers from every relevant field, the meeting provided the opportunity to crystallize misgivings and promoted a willingness to analyze data in a broader framework.

We sought to be inclusive and so invited a broad range of speakers that reflected all sides of the ongoing debate. The invited chairs were eminent cosmologists who guided the discussions and provided both a historical and physical perspective, drawing on their wide experience and backgrounds. As anticipated, we achieved lively arguments; we ensured that these were conducted in a collegial manner and they served to highlight many critical issues that need to be resolved to make progress.

We were particularly fortunate in the timing of this meeting, as Wendy Freedman was able to give the first public presentation of results from the ‘Chicago-Carnegie Hubble Program’. This applied new imaging from the James Webb Space Telescope to stars that can be used as standard candles: Cepheids and TRGB (tip of the red giant branch). However, a more recent addition to the armoury has come from the JAGB technique, which uses a particular colour selection (the ‘J region’) of Asymptotic Giant Branch stars [9]. The three determinations of $H_{0}$ are consistent with each other. Interestingly, the largest of the three is the Cepheid determination, which is consistent with the larger SH0ES figure, albeit with a slightly larger error. The combined determination is, however, centred at a figure well below the Cepheid value, reflecting the higher claimed precision of the TRGB and JAGB determinations: $(69.59\pm 1.58)\;km\;s^{-1}Mpc^{-1}$. This is barely consistent with the lower value from Planck, but in good agreement with the result from DESI. It will be interesting to see how this plays out with more data, but the novel JAGB technique is clearly a significant step towards achieving robust distances in cosmology. It is a matter of regret that we were not able to secure a write-up of these results as presented at the meeting, but details of the work can be found in [10].

## 2. What did we learn?

### (a) James Binney

Data have to be interpreted in a conceptual framework. Not so long ago, observations were interpreted on the understanding that the Earth lay at the centre of the cosmos. This conceptual model could account fully for the data, but from the Renaissance, it became accepted that the heliocentric model works better: the gross features of the data can be reproduced more naturally. As the data grew more precise, Kepler’s elliptical orbits had to be tweaked and the model lost some of its elegance, but in 1915, Einstein showed that the complexities of planetary motion *are* perfectly reproduced when the correct physics is applied to the heliocentric model. Ten years later, it emerged that the Sun is itself in orbit around one of billions of galaxies that are receding from one another.

If matter was conserved, the Universe must have come into existence remarkably recently, so many cosmologists preferred to hypothesize that matter is continuously created so the Universe can be eternal. The debate was resolved by the discovery of the CMB, which is a natural component of the Big Bang and hard to explain in a steady-state universe. The Universe had a finite age, but would it re-collapse? Studies of the fluctuations in the CMB, finally detected in 1992, established that the Universe is spatially flat and consequently will expand forever. General relativity (GR) connects spatial curvature to energy density and flatness requires a critical energy density. Galaxy rotation curves and clusters of galaxies had already pointed to most matter being non-baryonic dark matter. However, the peculiar velocities of galaxies were smaller than expected if galaxies and dark matter contained all the mass required to make the Universe spatially flat. Given the constraints on galaxy velocities, GR can account for the observed flatness only if the cosmic energy density is dominated by vacuum energy or, equivalently, a cosmological constant and the ΛCDM paradigm had arrived. The conclusion that distant supernovae are slightly fainter than would be expected without vacuum energy consolidated belief in ΛCDM.

This century has been an era of consolidation and refinement, analogous to the eighteenth century when astronomers sorted out details of the picture established by Newton standing on Kepler’s shoulders. While acknowledging the enormous success of the ΛCDM—see Efstathiou’s talk—several aspects make me uncomfortable.

1. The standard model of particle physics firmly establishes that the vacuum has an energy density that far exceeds that posited by cosmologists. The very existence of the Universe is incompatible with the right-hand side of Einstein’s field equations, comprising the expectation value ⟨T⟩ of the energy-momentum tensor T of matter fields. Evidently, at the level of quantum fluctuations, gravity does not couple to matter simply through ⟨T⟩. This being so, why should I believe GR’s prediction that flatness requires a certain critical energy density that lies orders of magnitude below the actual energy density?
2. Just as eighteenth-century cosmology was built around a simple and idealized dynamical model of the solar system, ΛCDM is built around a simple and idealized solution to Einstein’s equations. The solar system’s gravitational field was assumed to derive from the Sun and planets alone, while we now recognize extra-solar contributions can be important. Surely, the Universe will also prove more complex than many now suppose as we examine it in greater detail?
3. The ΛCDM model’s underlying solution describes a homogeneous, isotropic distribution of matter. The real Universe is lumpy and relating it to the ΛCDM solution is non-trivial. This is particularly true of velocity space—the solution specifies a zero velocity at each location and the Hubble parameter is defined by the way this zero varies from point to point. When the model is perturbed, matter acquires ‘peculiar’ velocities with respect to this zero, but we have no secure way of identifying this zero. In our own privileged case, we identify the zero with the frame in which the CMB has the smallest dipole, but since we observe the CMB through the distorting shroud of our Galaxy, even identification of the CMB ‘rest frame’ is not straightforward. Only one component of the velocity of an extra-galactic object is measurable and to determine the corresponding component of peculiar velocity, the object’s distance is required in addition to knowledge of the cosmic expansion rate $\dot{a}/a$, itself a function of time.
4. The model requires that the effects of lumpiness become negligible on the largest scales, but attempts to demonstrate this have failed. The assumption of underlying isotropy is fundamental to the standard model, but it is not a self-evident truth and it is hard to test empirically because observations cannot be made isotropically.

The aim of the meeting was to broaden the analysis of cosmological data from a narrow focus on adding significant figures to the reported values of the ΛCDM model’s six parameters to testing empirically the model’s key assumptions. Several reviews rightly reminded us of the wonderful precision with which the ΛCDM model accounts for observations of the CMB. As Peebles said, the model sets a high bar for any challenger theory. While much is beautifully explained, aspects of the data emerged that are not (yet) consistent with the model. Szapudi explained that the measured integrated Sachs–Wolfe effect is several times larger than expected; Secrest told us that the dipole in the angular distribution of radio galaxies is twice as large as is expected from the CMB dipole; Lopez reported the existence of two structures that are bigger than anything expected in the standard model; Watkins reported that the amplitude of the bulk flow increases rather than decreases with scale.

As we learn more about something, the complexity of the minimal model required to incorporate our knowledge invariably increases. Why should the Universe be an exception to this rule?

### (b) Roya Mohayaee

How does science progress? Does it progress through continuous remodelling, endless tightening of fits of parameters to improved data and incremental adjustment of approximations or through anomalies, groundbreaking insights and paradigm shifts? The most important discoveries in physics, GR and quantum mechanics, provide compelling evidence for the latter.

In the nineteenth century, Newtonian gravity was in crisis and astronomy was faced with an anomaly: the advance of the perihelion of Mercury. Le Verrier, a prominent astronomer of the time, had calculated the gravitational pull of every outer planet and determined that they would cause Mercury’s perihelion to advance by 527 arcseconds per century. This left a mere 43 arcseconds unaccounted for. Subsequently, solutions to the anomaly piled up: improved observations, more precise calculations, the oblateness of the Sun and even tweaking of Newton’s inverse square law were invoked to account for the missing 43 arcseconds. Le Verrier himself proposed that an intramercurial planet, Vulcan, could resolve this anomaly. Le Verrier’s Vulcan was widely accepted by the community and even by the Royal Astronomical Society. Why? Well, Newton’s theory of gravity could account for just about everything in celestial mechanics. Moreover, Le Verrier was highly respected and had earlier predicted the existence of Neptune by using fluctuations in the orbit of Uranus. For decades, reputed astronomers hunted for Vulcan and a few even claimed to have found it. It was only a paradigm shift that finally resolved this anomaly. Einstein made explaining Mercury’s precession a primary goal and even rejected his initial draft of GR on the grounds that it did not account for the missing 43 arcseconds. This anomaly now provides an iconic test of GR.

Quantum mechanics was born similarly. In the early twentieth century, Maxwell’s beautiful theory led to a notorious anomaly, the ‘ultraviolet catastrophe’, which is reminiscent of our cosmological constant problem. Application of the ideas of Boltzmann to the electromagnetic field in a cavity led Rayleigh and Jeans to a formula for the spectral content of black-body radiation that agreed with measurements at low frequencies but predicted that the radiation’s intensity would diverge at high frequencies. Changes to classical theory were proposed and rejected for years until the groundbreaking work of Max Planck. He found a simple formula that fitted the data at all frequencies and then sought a mechanical model that would predict this formula via Boltzmann’s apparatus. He discovered that the cavity’s normal modes would reproduce the fitting formula if they absorbed and emitted energy only in discrete amounts. Thus, was quantum mechanics born: a paradigm shift that propelled us into a whole new era in physics.

Are we living in a time that carries a paradigm shift? The ΛCDM model provides a good fit to observations and its apparent success is so overwhelming that one often forgets that two building blocks of this model are ‘missing’! One could argue that dark matter only interacts through gravity and hence is difficult to detect. What about the other 70% of cosmic energy? The cosmological constant is unquestionably the biggest challenge of theoretical physics today. A homogeneous and isotropic model of the Universe is simple, easy to solve and high-yielding. It is, however, disconcerting that our two ‘standard designs’, one of cosmology and one of particle physics, are antipoles: the former is a simple model and the latter is a fundamental and hugely complex theory.

Can the anomalies that have been haunting ΛCDM*—*e.g. the anomalous quasar and radio dipole, which now stand at over 5σ, the Hubble tension and the persisting non-convergence of the bulk flow—show us the way towards a fundamental theory of cosmology? Or, as Jim Peebles puts it, ‘The standard ΛCDM cosmology passes demanding tests that establish it as a good approximation to reality. It is incomplete, with open questions and anomalies, but the same is true of all our physical theories. The anomalies in the standard cosmology might guide us to an even better theory. It has happened before’.

### (c) John Peacock

Is it really possible that a standard cosmological model has come into being that is basically correct in its description of the constitution of the Universe? It would be remarkable that this singular event can have occurred during the lives of the current generation of astronomers—and so we should be open to the possibility that cosmology in 2074 might have changed by as much as our ideas have evolved between 1974 and the present.

And yet, having worked in the subject for more than four decades, my experience is that radical change is not that frequent. After the flurry of activity around nucleosynthesis in the 1970s and false reports of massive neutrinos in 1980, the community had focused by 1982 on a simple model of adiabatic fluctuations in CDM. The big early mistake was to reject the idea of a non-zero Λ, leading to a 1980s-era ‘standard’ CDM model that had a critical matter density. Then, as now, many articles were written pointing out observations that failed to chime with the standard picture, leading to its downfall in the 1990s. The contrarian 1980/1990s evidence often pointed in various ways towards a subcritical matter density, from which one could infer the need for Λ, since a heavily open model would violate limits on small-scale CMB anisotropies. At the end of the 1990s, the more direct evidence for Λ from SNe added weight to this case and the community accepted ΛCDM as the standard model overnight.

This historical revolution feels very different from the present situation. The 1980s had a (wrong) standard model, but also ΛCDM as a well-motivated competitor—so that tensions with the standard model were frequently viewed in terms of this choice between alternatives. At this meeting, we have heard of a number of challenges to the current standard model, but it is hard to see that they point in a consistent direction. In particular, there are some issues with different expansion histories, which can be reconciled by assuming the evolution of dark energy; separately, there are various arguments for large-scale anisotropies in velocity or number density, though the anisotropy direction seems to be different in each case.

It is therefore reasonable to wonder if these diverse problems reflect no more than imperfectly understood datasets. We should recall that cosmological data have improved out of all recognition in the more than 25 years that ΛCDM has been the standard model. This model had an excellent chance to fail when WMAP reported its transformational CMB results in 2003, but it failed to grasp the opportunity. Today the Planck CMB results provide one of the most beautiful examples of agreement of data and theory in all of the physical sciences. However, the formal precision of such datasets is now challengingly high—is it realistic to assume that they are perfect? Thus, when Planck and DESI argue over whether $\Omega_{m}$ is 0.315 or 0.295, I personally find it much more probable that this tiny difference reflects remaining small systematics in one or the other dataset, rather than a bizarre evolution of dark energy.

Finally, the other problem with so many of the discrepancies highlighted at this meeting is that there is a danger of forgetting how well so much of the standard model works. For example, anyone who is interested in anomalous velocity fields should inspect fig. 1 of Davis & Nusser [11] and note the exquisite agreement between the pattern of peculiar velocities and predictions from LSS. This is at distances up to 150 Mpc; is it plausible that the standard model happens to fail at larger scales, just at the point where the velocity data cease to be of high S/N?

In short, I am very glad that we made a concerted effort to examine challenges to the standard model in this meeting, but I see nothing that really convinces me that its demise is imminent. I make this statement knowing that future historians of science may well enjoy heaping scorn on me for lack of imagination in failing to see the coming revolution.

### (d) Subir Sarkar

The community overwhelmingly believes in the standard ΛCDM model and much is made of its success in fitting a wide variety of observations with just a few parameters. What tends to be forgotten is that one of these parameters is the cosmological constant Λ, which is *inferred* to have a value of $O(H_{0}^{2})$. Here $H_{0}$, the present value of the Hubble expansion rate is neither fundamental nor a constant. It enters, however, into *every* cosmological measurement and it is easy to see how its value is imprinted on Λ via the ‘Cosmic sum rule’ $\Omega_{m}+\Omega_{k}+\Omega_{\Lambda }=1$, which is just a restatement of the Friedmann–Lemaitre equation that the ten coupled Einstein equations reduce to for the assumed maximally symmetric FLRW metric and the assumed ‘ideal fluid’ matter content of the Universe. As $\Omega_{\Lambda }\equiv \Lambda /3H_{0}^{2}$, it is evident that Λ is forced to take on such a value when it is extracted using measurements of $\Omega_{k}≃0$ (CMB), $\Omega_{m}∼0.3$ (clusters, BAO) and $0.8\Omega_{m}-0.6\Omega_{\Lambda }∼-0.2$ (SNe Ia)—which together imply that $\Omega_{\Lambda }∼0.7$. Out of this simplistic argument comes the profound conclusion that the Universe is dominated today by the energy of the quantum vacuum if we choose to interpret Λ as an energy density $\rho_{\Lambda }=\Lambda /8\pi G_{N}$. In the natural units used by particle physicists, $H_{0}∼10^{-42}$ GeV while $M_{\mathrm{Pl}}\equiv (8\pi G_{N}{)}^{-1/2}∼10^{19}$ GeV, thus this vacuum energy has a scale of $∼10^{-12}$ GeV which has no place in the Standard Model—the successful quantum field theory of the electromagnetic, weak and strong interactions. The unsolved cosmological constant problem at the vexed boundary between quantum field theory and classical gravity is further exacerbated by the suggestion that the vacuum energy that gravitates is not even exactly zero but takes on a tiny value that is at least 60 orders of magnitude smaller than might be reasonably expected in the standard model, which is experimentally established to hold at least up to the electroweak unification scale of $O(10^{3})$ GeV. It has long been recognized that the energy of the quantum vacuum cannot couple to gravity (as is expected for all forms of energy in GR) since in that case the Universe would not have survived past the early epoch of $∼10^{-12}$ s when it cooled down to the electroweak scale. And yet we are here today, 14 billion years later, inferring a cosmological constant determined by the *present* rate of expansion. The highly contrived nature of this deduction becomes even more evident when we note the enormous fine-tuning that is required—between the cosmological constant multiplying the metric tensor on the left-hand side of Einstein’s equations (reflecting its invariance under local coordinate transformations) and the super-renormalizable zero-point vacuum energy from the energy-momentum tensor on the right-hand side when it is extended to include quantum fields and not just ‘dust’ or radiation. These two terms do not ‘know’ about each other (except in very special backgrounds); nevertheless, they must cancel if the Universe is to exist in its present form. Yet we know of no underlying symmetry that can ensure this. Given this formidable challenge, it seems even more perverse to argue that not only is there such a miraculous cancellation, but that it is not quite exact and there is a residual Λ of $O(H_{0}^{2})$ such that approximately 70% of the Universe today is dark energy. Should we not be examining just how we got ourselves into this mess, rather than believing that we have a ‘standard’ model that is moreover ‘simple’?

The clue must lie in the (over)simplification of the large-scale Universe that is embodied in the adoption of the FLRW metric, that is, the assumption that it is isotropic and homogeneous when averaged on large scales. Even as our observational abilities have improved dramatically, we are still using this century-old model to interpret data (and concluding that there is ‘concordance’ with the ΛCDM model). Whereas this assumption was justified a century ago when there was hardly any data, little attempt is being made even today to check that the data are consistent with this crucial assumption. A case in point is the beautiful data from DESI, which were presented at this meeting by Nathalie Palanque–Delabrouille—the millions of exquisitely precise redshift measurements over a reasonably large patch of the sky are nevertheless being interpreted assuming FLRW; no statement is made about whether the observed BAO signal is indeed the same in all the directions surveyed. This in spite of the strong evidence presented earlier by Nathan Secrest that the distribution of matter is *not* isotropic in the reference frame in which the CMB is believed to be isotropic, to which all observations are boosted (to cancel out the CMB dipole) before applying the Friedmann–Lemaitre equation. Mohamed Rameez demonstrated that the acceleration inferred from the Hubble diagram of Type Ia supernovae is in fact *anisotropic* implying that it cannot be due to Λ. It is probably due to our being ‘tilted’ observers, embedded as we are in an anomalously deep and fast bulk flow, evidence for which was presented by Richard Watkins. All these observations are quite contrary to the expectations in the ΛCDM model and moreover, direct evidence for the existence of Λ, e.g. the late Integrated Sachs–Wolfe effect, is still a vexed issue as Istvan Szapudi discussed (table 1).

**Table 1.** List of speakers (Programme).

speaker	organisation	talk title	paper
Jim Peebles	Princeton University	Status of the ΛCDM theory: supporting evidence and anomalies	yes
George Efstathiou	University of Cambridge	Challenges to the ΛCDM cosmology	yes
Wendy Freedman	University of Chicago	New JWST results: is the current tension in H_0 signalling new physics?	no
Alexandra Amon	Princeton University	Weak lensing with a billion galaxies: Testing the cosmological model	no
Mathew Madhavacheril	University of Pennsylvania	Assessing the growth of structure over cosmic time with CMB lensing	yes
István Szapudi	University of Hawai’i	The ISW puzzle	ye
Nathan Secrest	US Naval Observatory, Washington	The Ellis–Baldwin test	yes
Chris Clarkson	Queen Mary, University of London	The cosmological priniciple and the kinematic dipole	no
Alexia Lopez	University of Central Lancashire	Investigating ultra-large large-scale structures: Potential implications for cosmology	yes
Konstantinos Migkas	Leiden University	Galaxy clusters as probes of cosmic isotropy	yes
Richard Watkins	Willamette University, Oregon	Challenges to the standard cosmological model from large-scale bulk flow estimates	yes
Mohamed Rameez	Tata Institute of Fundamental Research, Mumbai	Anisotropy in the cosmic acceleration inferred from supernovae	yes
Gia Dvali	Ludwig Maximilian University and Max Planck Institute, Munich	S-matrix constraints on dark energy	no
Oliver Philcox	Columbia University and Simons Foundation	Could sample variance be responsible for the parity-violating signal seen in the BOSS galaxy survey?	yes
Nathalie Palanque-Delabrouille	Lawrence Berkeley National Laboratory	Future directions in cosmology	yes
Chairs + Organisers		Panel discussion	

All talks are uploaded on: https://youtube.com/playlist?list=PLg7f-TkW11iV7zMH7UiawArnTvrar6ABU&si=vwwL2SpS7YNiAQn6

The last standard model of cosmology, owing to Ptolemy and Aristotle, held sway for two millennia before it was overthrown for lack of a physical foundation. The current standard model has been around for only a couple of decades. Let us see how long it lasts!

## 3. Conclusion

The above text was perhaps typical of the reactions of participants at the meeting, where the role of Bayes' theorem loomed large. No scientific analysis is ever definitive or free from ’unknown unknowns’, so there can be a tendency to cling to prior beliefs until a sufficiently large pile of evidence finally forces these to be abandoned. Different individuals place the critical bar for the evidence in different places and the present editors are no exception: some are motivated by the simplicity and long record of success of the ΛCDM model to be highly sceptical of claimed disproofs, while others feel on fundamental grounds that the model is unlikely to be true. Certainly, there is plenty of discrepant evidence available to back up the latter view. But a shift to a new paradigm requires an alternative theory: the significance of the anomalous precession of Mercury lay not just in the fact that it was in conflict with Newtonian theory but that the observation was inevitable in GR—a physical theory for which powerful fundamental principles drove a framework that made rather robust predictions. It is fair to say that cosmology currently lacks a beyond-ΛCDM theory of such stature and until this emerges, there will probably remain a degree of puzzled disagreement as to the meaning of observational anomalies. However, the subject does not stand still: the data will improve and theorists will continue their efforts. So, in due course, we can expect the mist to clear, but for the present, we are content to have been able to present a summary of where things stand.

## Data Availability

This article has no additional data.
